# CyNetSVM: A Cytoscape App for Cancer Biomarker Identification Using Network Constrained Support Vector Machines

**DOI:** 10.1371/journal.pone.0170482

**Published:** 2017-01-25

**Authors:** Xu Shi, Sharmi Banerjee, Li Chen, Leena Hilakivi-Clarke, Robert Clarke, Jianhua Xuan

**Affiliations:** 1 Department of Electrical and Computer Engineering, Virginia Polytechnic Institute and State University, Arlington, Virginia, United States of America; 2 Department of Pathology, Johns Hopkins University School of Medicine, Baltimore, Maryland, United States of America; 3 Departments of Oncology, Lombardi Comprehensive Cancer Center, Georgetown University, Washington, DC, United States of America; University of Texas at San Antonio, UNITED STATES

## Abstract

One of the important tasks in cancer research is to identify biomarkers and build classification models for clinical outcome prediction. In this paper, we develop a CyNetSVM software package, implemented in Java and integrated with Cytoscape as an app, to identify network biomarkers using network-constrained support vector machines (NetSVM). The Cytoscape app of NetSVM is specifically designed to improve the usability of NetSVM with the following enhancements: (1) user-friendly graphical user interface (GUI), (2) computationally efficient core program and (3) convenient network visualization capability. The CyNetSVM app has been used to analyze breast cancer data to identify network genes associated with breast cancer recurrence. The biological function of these network genes is enriched in signaling pathways associated with breast cancer progression, showing the effectiveness of CyNetSVM for cancer biomarker identification. The CyNetSVM package is available at Cytoscape App Store and http://sourceforge.net/projects/netsvmjava; a sample data set is also provided at sourceforge.net.

## Introduction

Genes usually work collaboratively as modules, networks or pathways, and different modules can interact with each other to take effect [1]. The nature of complex interactions makes it difficult to elucidate biological mechanisms from individual gene-based approaches [2]. Several approaches have been proposed to identify gene sets, networks or pathways involved in cancers, e.g., gene set enrichment [3], network-constrained linear regression [4] and mutual information-based network scoring [5]. More recently, NetSVM [6] has been developed to identify predictive biomarkers (i.e., gene networks) by integrating gene expression data and protein-protein interactions (PPI) data. Specifically, the NetSVM approach takes into account the dependency of genes in a network and incorporates it into the prediction scheme of support vector machine (SVM) for improved performance in identifying network biomarkers (as previously demonstrated in [6]).

In this paper, we present a Cytoscape [7] app, called CyNetSVM, that implements the NetSVM method, an integrated approach to predict clinical outcome of patients and to identify biologically meaningful networks. The core (analytic) program is implemented in Java so as to analyze large-scale biomedical data efficiently. To further support the ease of use of NetSVM, a user-friendly graphical user interface (GUI) is developed. The data and necessary options can be easily set through the GUI. Both the core analytic program and GUI are integrated with Cytoscape using Cytoscape application program interface (API). The CyNetSVM app not only provides the prediction performance (i.e., sensitivity and specificity) but also generates a network view of the identified biomarkers in Cytoscape. We first use a simulation study to show the correctness of implementation and the advantage of incorporating network information. To demonstrate the capability of CyNetSVM in real biomedical applications, we further use the CyNetSVM app to analyze breast cancer data for clinical outcome prediction and network biomarker identification. The experimental result demonstrates that CyNetSVM can provide high sensitivity and specificity for clinical outcome prediction. Furthermore, functional analyses of the identified gene networks show a significant enrichment in breast cancer-related signaling pathways.

## Materials and Methods

**Fig 1:**
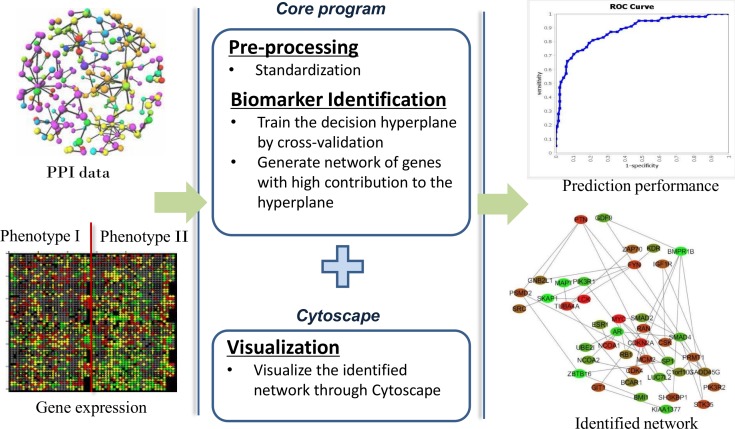
An overview of the CyNetSVM app.

An overview of the CyNetSVM package is shown in Fig 1. The core program of the CyNetSVM app is implemented in Java and integrated with Cytoscape using Cytoscape API. After input data is collected (*i*.*e*. protein-protein interaction (PPI) data and gene expression data), the core program first pre-processes the data through standardization and then identifies the networks from the processed data. Once the core program completes, the gene network is created, and the node color is set based on the *log2* fold change between the two phenotypes. Along with the network, CyNetSVM also reports the sensitivity, specificity, ROC curve and AUC values for the classification.

### The NetSVM Method

NetSVM [6] is a computational method to predict clinical outcome and identify network biomarkers by integrating gene expression data and PPI data. As an extension of the conventional support vector machine (SVM), NetSVM also exploits the decision hyperplane to predict the clinical outcome of patients. The gene dependency in a network is incorporated as a constraint upon the objective function of conventional SVM. The network constraint is formulated by a Laplacian matrix, which is calculated from PPI data. By utilizing the smoothing property of the Laplacian matrix, genes in a network tend to have a similar contribution to the decision hyperplane. The objective function of NetSVM can be rewritten in the same form as that of conventional SVM by transforming the hyperplane parameters or rotating the hyperplane. Therefore, the optimization problem of NetSVM can be solved as that of conventional SVM, and the solution, i.e., the hyperplane, can then be rotated back. The final identified network consists of the genes with higher contribution to the hyperplane.

### Software Implementation

**Fig 2:**
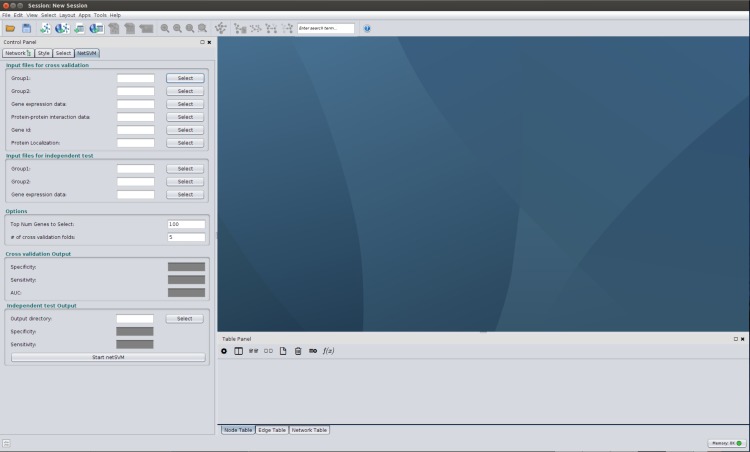
Screenshot of the CyNetSVM app.

The CyNetSVM package has been implemented in Java as a Cytoscape app for network biomarker identification. A screenshot of the CyNetSVM app is shown in Fig 2. We designed a user-friendly GUI in the left panel for users to access to the plugin. The following input files are needed (described in Table 1)—gene expression data in standard GCT format, protein-protein interaction (PPI) data (formatted as tab-separated values (TSV) format) and class label indices of samples. Typically, gene expression data and PPI network data contain a large number of genes or proteins. In many cases, users are only interested in a selective set of genes, such as genes of breast cancer pathways. For CyNetSVM, users can provide a subset of genes selected from the original gene list. The subset of PPI network only with these genes will be extracted to perform the analysis. To tune the weight of network constraint, we apply cross-validation to find the parameters that provide the best accuracy. Users can set the number of folds for the cross-validation. To visualize the identified network, users need to determine the size of the network, which is the same as setting a threshold to select top-ranked genes. Improved visualization of the identified network can be obtained by providing a file containing the mapping between gene symbol and protein’s cellular location. The genes shown in the network will be grouped by the cellular location of proteins.

**Table 1:** Input Data of CyNetSVM.

Data	Format	Description
Protein-protein interaction data	TSV	Protein interaction networks
Gene expression data	GCT	Microarray gene expression data
Group 1 index	TSV	Group 1 (e.g., early recurrence) (value = 1)
Group 2 index	TSV	Group 2 (e.g., late recurrence) (value = 2)
Gene id	TSV	Gene list of interest
Gene product location	TSV	File containing gene Entrez ID, gene symbol, gene product location, and participated pathways in the cell

When running the CyNetSVM app, the GUI will pass all the input data and options to the core program. The class diagram of the GUI component is shown in S1 Fig. The classes of NetSVMParameterPanel and NetSVMDataPanel are responsible for collecting the parameters and data files needed to run the plugin, respectively. The NetSVMRunPanel class is designed to act as an interface bridging the input data and the core analytic program. Data preprocessing, such as standardization, will be performed on the gene expression data. Cross-validation will then start with the number of folds set by the user. As a final step, the specificity, sensitivity and the area under the receiver operating characteristic (ROC) curve (AUC) will be calculated and reported. Further, the CyNetSVM app will generate a network view of the identified biomarkers in Cytoscape.

Since Cytoscape uses the OSGi architecture (https://www.osgi.org), CyNetSVM has been packaged as a bundle in Cytoscape. S2 Fig shows the class diagram of the CyNetSVM bundle app. The core program of CyNetSVM is implemented as a Java program that can be run through Cytoscape API. The CyActivator class is the Activator for the bundle, trigger every time the bundle is started or stopped. To run the package, the bundle needs to be loaded in the OSGi container and started. Additionally, the package uses CreateNetwork (a Cytoscape built-in class) to obtain the results from the core program; it also uses CyNetworkFactory to construct a network from the identified genes and CyNetworkManager to display (show) the network.

## Results and Discussion

### Simulation Data

We first compared CyNetSVM with NetSVM (implemented in MATLAB) and conventional SVM using simulation data to prove the correctness of our implementation and demonstrate the improvement of performance with network information incorporated. The simulation data were generated on a breast cancer-related network with 584 genes and 2280 nodes following the same strategy used in [6]. For each phenotype, we generated 100 samples for both training and testing data. To evaluate the performance under different levels of noise, we simulated 11 scenarios with different signal-to-noise ratios (SNR) ranging from -10 dB to 10 dB. For each scenario, we generated 100 simulation data sets to evaluate the variance of performance. Table 2 shows the accuracy of phenotype prediction and the area under the ROC curve (AUC) for network identification. It can be seen that the performance of CyNetSVM and NetSVM are very close, which shows the correctness of our implementation. Note that the minor difference of the performance between CyNetSVM and NetSVM is mainly caused by the stochasticity of the cross-validation procedure. Furthermore, the significant improvement of network identification of CyNetSVM and NetSVM compared with SVM demonstrates the importance of incorporating network information.

**Table 2:** Means and standard deviations of accuracy for phenotype prediction and AUC for network identification on simulation data with different SNR.

SNR (dB)	Phenotype prediction (accuracy)			Network identification (AUC)		
	CyNetSVM	NetSVM	SVM	CyNetSVM	NetSVM	SVM
10	1.00 ± 0.00	1.00 ± 0.00	1.00 ± 0.00	0.86 ± 0.04	0.85 ± 0.03	0.76 ± 0.04
8	1.00 ± 0.00	1.00 ± 0.00	1.00 ± 0.00	0.83 ± 0.05	0.84 ± 0.06	0.76 ± 0.04
6	1.00 ± 0.00	1.00 ± 0.00	1.00 ± 0.00	0.83 ± 0.04	0.84 ± 0.04	0.77 ± 0.03
4	1.00 ± 0.00	1.00 ± 0.00	1.00 ± 0.00	0.83 ± 0.06	0.84 ± 0.06	0.77 ± 0.03
2	1.00 ± 0.00	1.00 ± 0.00	1.00 ± 0.00	0.83 ± 0.05	0.83 ± 0.06	0.76 ± 0.03
0	0.99 ± 0.01	0.99 ± 0.01	0.99 ± 0.01	0.83 ± 0.04	0.83 ± 0.04	0.77 ± 0.04
-2	0.98 ± 0.02	0.98 ± 0.02	0.99 ± 0.01	0.81 ± 0.03	0.80 ± 0.04	0.74 ± 0.05
-4	0.91 ± 0.02	0.91 ± 0.03	0.91 ± 0.02	0.79 ± 0.04	0.79 ± 0.07	0.72 ± 0.02
-6	0.85 ± 0.03	0.85 ± 0.03	0.83 ± 0.04	0.79 ± 0.06	0.79 ± 0.06	0.71 ± 0.03
-8	0.77 ± 0.06	0.78 ± 0.06	0.78 ± 0.06	0.79 ± 0.06	0.79 ± 0.07	0.72 ± 0.04
-10	0.71 ± 0.06	0.70 ± 0.05	0.71 ± 0.05	0.70 ± 0.05	0.71 ± 0.05	0.68 ± 0.03

### Network Identification from Breast Cancer Data

**Fig 3:**
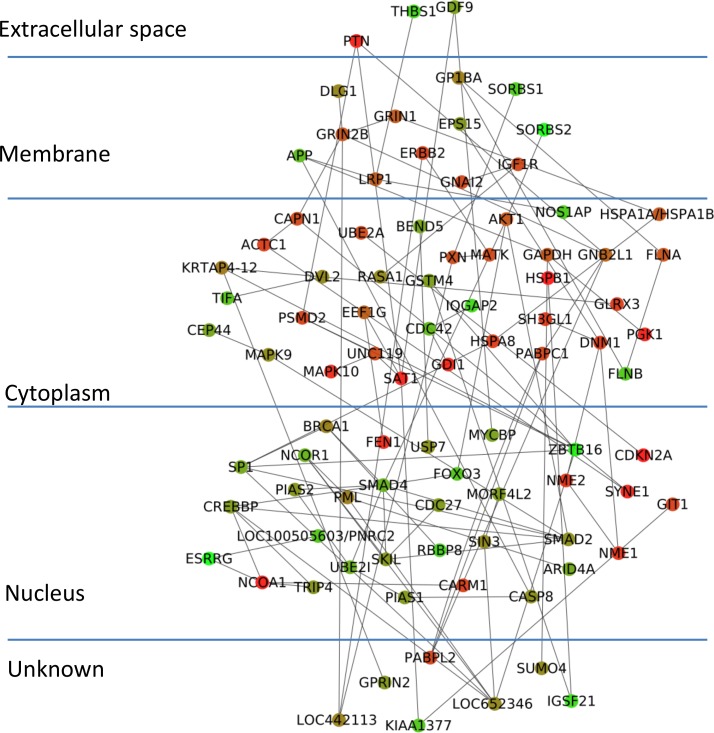
Network identified from Loi *et al*. data.

**Fig 4:**
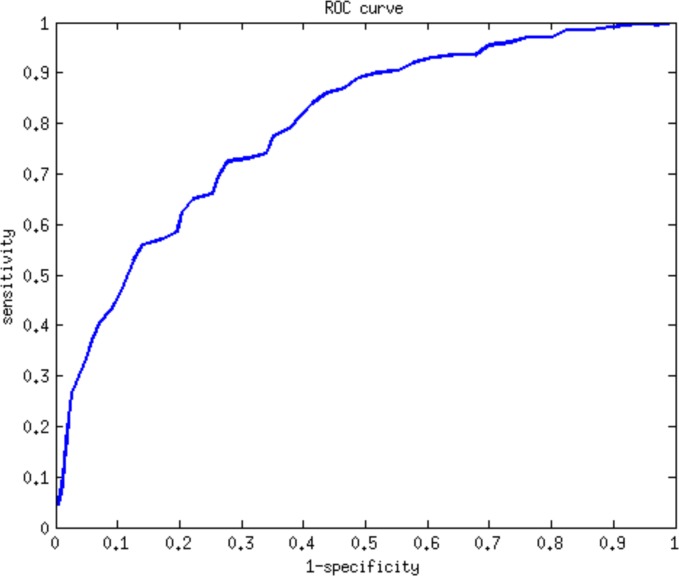
ROC curve of the classification of patients in Loi *et al*. data.

To demonstrate the effectiveness of CyNetSVM for real biomedical applications, the CyNetSVM app was used to analyze a breast cancer gene expression dataset (Loi *et al*. data) [8]. The samples were divided into two groups, ‘early recurrence’ and ‘late recurrence,' separated by six years in survival time. We obtained 20 samples in the ‘early recurrence’ group and 27 samples in the ‘late recurrence’ group. In this study, we used the whole PPI network from the HPRD database [9] (9673 nodes and 40563 edges after mapping to the microarray platform) to evaluate the performance. We further applied the Bagging Markov Random Field (BMRF) method [10, 11] on both networks and obtained networks of 484 genes and 2096 edges to start with the analysis. The program completed the network analysis less than 10 seconds with 5-fold cross-validation. The identified network with top 100 genes is shown in Fig 3. We further applied the DAVID [12] functional annotation tool (https://david-d.ncifcrf.gov/) on the identified genes. The genes in the network are significantly enriched in breast cancer-related pathways such as FOXO signaling pathway [13], MAPK signaling pathway [14], Ras signaling pathway [15], TGF-Beta signaling pathway [16], Estrogen signaling pathway [17], Wnt signaling pathway [18] and ErbB signaling pathway [19]. The detailed functional annotation results are shown in Table 3. The p-value was calculated using the genes measured in the PPI data as the background genes. For the prediction of recurrence status (i.e., ‘early recurrence’ or ‘late recurrence’), CyNetSVM achieved a sensitivity of 0.73 and a specificity of 0.72. We also set a different threshold for the absolute weight of gene to conduct a ROC study of the prediction. As shown in Fig 4, the AUC value is 0.80. The experimental results show that the CyNetSVM app can be used as an effective tool for network biomarker identification.

**Table 3:** Functional enrichment of genes identified from Loi *et al*. data in signaling pathways and associated p-values.

Pathway	Genes	P-value
FOXO signaling pathway	AKT1,CREBBP,SMAD2,SMAD4,FOXO3,IGF1R,MAPK10,MAPK9,PLK1,USP7	1.1E-6
MAPK signaling pathway	AKT1,RASA1,CDC42,FLNA,FLNB,HSPA1B,HSPA8,HSPB1,MAPK10,MAPK9	1.2E-4
Ras signaling pathway	AKT1,RASA1,CDC42,GRIN1,GRIN2B,IGF1R,MAPK10,MAPK9	1.1E-3
TGF-Beta signaling pathway	CREBBP,SMAD2,SMAD4,SP1,THBS1	1.3E-3
Estrogen signaling pathway	AKT1,GNAI2,SP1,HSPA1B,HSPA8	2.3E-3
Wnt signaling pathway	CREBBP,SMAD4,DVL2,MAPK10,MAPK9	6.9E-3
ErbB signaling pathway	AKT1,ERBB2,MAPK10,MAPK9	1.2E-2

### Network Analysis Using METABRIC Data

**Fig 5:**
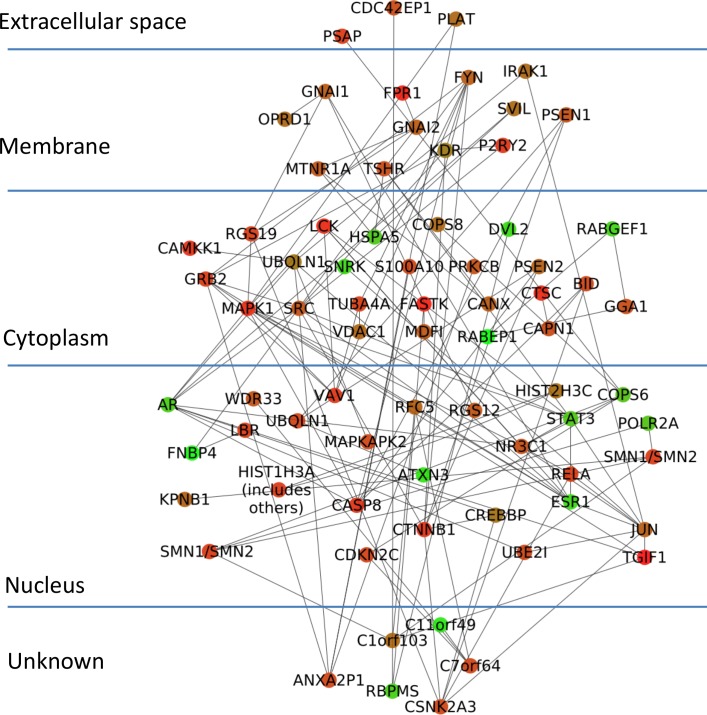
Network identified from METABRIC discovery data.

**Fig 6:**
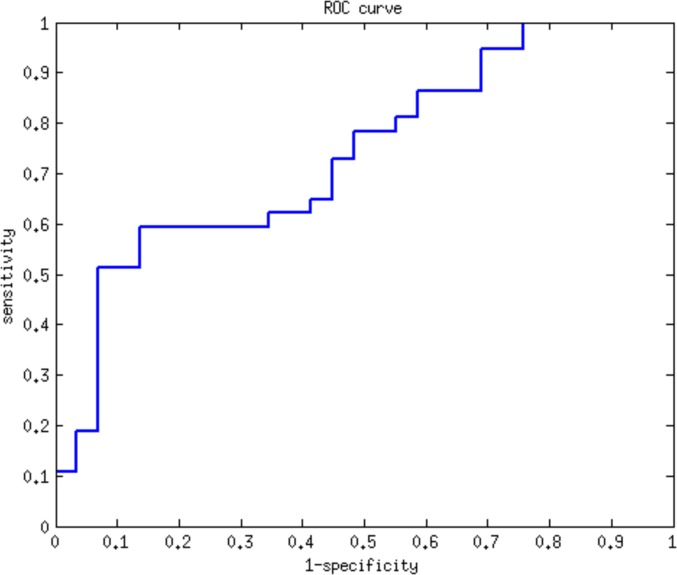
ROC curve of the classification of patients in METABRIC validation data.

We further applied CyNetSVM to the METABRIC data [20] to demonstrate the effectiveness of network analysis on independent data sets. The METABRIC data were divided into a discovery dataset (997 samples) and validation dataset (989 samples). The samples were further selected by ER status (ER positive), treatment method (hormone treatment) and survival status (death), resulting in 208 samples in the discovery dataset and 220 samples in the validation dataset. The samples were further classified into ‘early recurrence’ group (< 3 years) and ‘late recurrence’ (> 9 years and < 12 years) by survival time. Finally, the discovery dataset consisted of 41 samples in the ‘early recurrence’ group and 44 samples in the ‘late recurrence’ group; the validation dataset consisted of 37 samples in the ‘early recurrence’ group and 29 samples in the ‘late recurrence’ group. In this study, we also used the whole PPI network from the HPRD database. After mapping the genes to the microarray platform, we obtained 9579 nodes and 40281 edges in the network. We further applied the BMRF method onto the network to identify subnetworks with 597 nodes and 2828 edges. Based on the network, CyNetSVM took about 10 seconds to train on the discovery data and test on the validation data. Fig 5 shows the identified networks with top 100 genes. We further used the DAVID functional analysis tool to analyze the genes in the network. The results showed that the genes are significantly enriched in breast cancer-related pathways such as Estrogen signaling pathway [17], Ras signaling pathway [15], ErbB signaling pathway [19], MAPK signaling pathway [14], TGF-Beta signaling pathway [16], Wnt signaling pathway [18] and FOXO signaling pathway [13]. Table 4 lists the genes and corresponding significance level in signaling pathways. As the reproducibility of biomarker identification has been a challenging problem in the field [21], the genes identified from the Loi *et al*. data and the discovery data are quite different, with only seven genes (i.e., CREBBP, DVL2, AKT1, GNAI2, UBE2I, CAPN1 and CASP8) in common. However, enriched signaling pathways are consistent (as we can see from Tables 3 and 4), showing a convergent point of the identified networks at the functional level. Regarding recurrence status prediction, CyNetSVM achieved AUC of 0.7372 with sensitivity of 0.6216 and specificity of 0.6552. The ROC curve is shown in Fig 6.

**Table 4:** Functional enrichment of genes identified from the discovery dataset in signaling pathways and associated p-values.

Pathway	Genes	P-value
Estrogen signaling pathway	AKT1,GNAI1,GNAI2,JUN,SRC,ESR1,GRB2,MAPK1	5.6E-6
Ras signaling pathway	AKT1,RELA,GRB2,KDR,MAPK1,NF1,PDGFB,PRKCB,RGL1	2.5E-4
ErbB signaling pathway	AKT1,JUN,SRC,GRB2,MAPK1,PRKCB	3.2E-4
MAPK signaling pathway	AKT1,JUN,RELA,GRB2,MAPK1,MAPKAPK2,NF1,PDGFB,PRKCB	5.2E-4
TGF-Beta signaling pathway	CREBBP,TGIF1,BMP6,GDF6,MAPK1	1.3E-3
Wnt signaling pathway	CREBBP,JUN,CTNNB1,DVL2,PSEN1,PRKCB	1.4E-3
FOXO signaling pathway	AKT1,CREBBP,GRB2,MAPK1,STAT3	9.0E-3

### Scalability

Given the Loi *et al*. dataset [8], we have also evaluated the scalability of CyNetSVM by measuring the computational time on networks with a different number of nodes and edges up to the whole HPRD PPI network. The results are shown in Table 5 (as tested on a DELL PC Workstation (Precision T7600) with 2.9 GHz Intel Xeon CPU and 46 GB memory). It can be seen from the table that the CyNetSVM app can complete the identification process within 90 seconds on a relatively large network with 1000 nodes. The fast speed of the CyNetSVM app makes it an efficient tool to help identify network biomarkers and visualize the network in Cytoscape. We also measured the computational performance on networks with the same number of nodes (1000) but with different average node degrees. The results show that the computational time is robust against the average node degree. Theoretically, the increase of average node degree will not lead to a significant increase of computational time. The most time consuming calculation in the NetSVM method is the matrix decomposition of the Laplacian matrix. The scale of the Laplacian matrix is determined only by the size of nodes. For example, extremely large networks (i.e., Number of nodes > 5000) will significantly increase the computational burden of the app while dealing with matrix decomposition with dimension over 5000×5000. Also, directly applying CyNetSVM on overwhelmed large networks will degrade the performance. In dealing with a large network, we recommend users to construct a disease-related gene list from databases such as GO database [22] and KEGG pathways [23] and input the gene list to the app. If the gene list is not available, users can apply methods such as jActiveModule [24] and BMRF [10, 11] to first select potential disease-related genes and networks as input.

**Table 5:** Computational time of the CyNetSVM app as tested with different network sizes and cross-validation folds.

No. of nodes	No. of edges	Average node degree	Cross-validation folds	Time (sec)
100	143	2.86	5	3.1
100	143	2.86	10	3.7
300	553	3.69	5	4.5
300	553	3.69	10	5.4
500	2162	8.65	5	8.3
500	2162	8.65	10	9.2
1000	2181	4.36	5	60.1
1000	2181	4.36	10	64.3
1000	3539	7.20	5	60.9
1000	3539	7.20	10	65.2
1000	4919	9.84	5	59.7
1000	4919	9.84	10	64.8
2545	15094	11.86	5	1856.3
2545	15094	11.86	10	1874.9
5000	19207	7.68	5	2980.3
5000	19207	7.68	10	2998.8
9673	40563	8.39	5	21653.5
9673	40563	8.39	10	21706.3

## Conclusions

The CyNetSVM app is a software tool that can be used to identify biologically meaningful network biomarkers from PPI network and gene expression data. Equipped with user-friendly GUI, computationally efficient core program (implemented in Java) and network visualization capability of Cytoscape, the CyNetSVM app can be applied to large-scale real biomedical data to effectively identify biomarkers and conveniently visualize biomarker networks.

## Supporting Information

S1 Fig The class diagram of the CyNetSVM GUI. (PDF)

S2 Fig The class diagram of the CyNetSVM bundle application. (PDF)
