# Supplementary figures and images for: CyNetSVM: A Cytoscape App for Cancer Biomarker Identification Using Network Constrained Support Vector Machines
Source: PLoS One. 2017 Jan 25;12(1):e0170482. doi: 10.1371/journal.pone.0170482 (PMC5266326; doi:10.1371/journal.pone.0170482)

**S1 Fig. The class diagram of the CyNetSVM GUI**

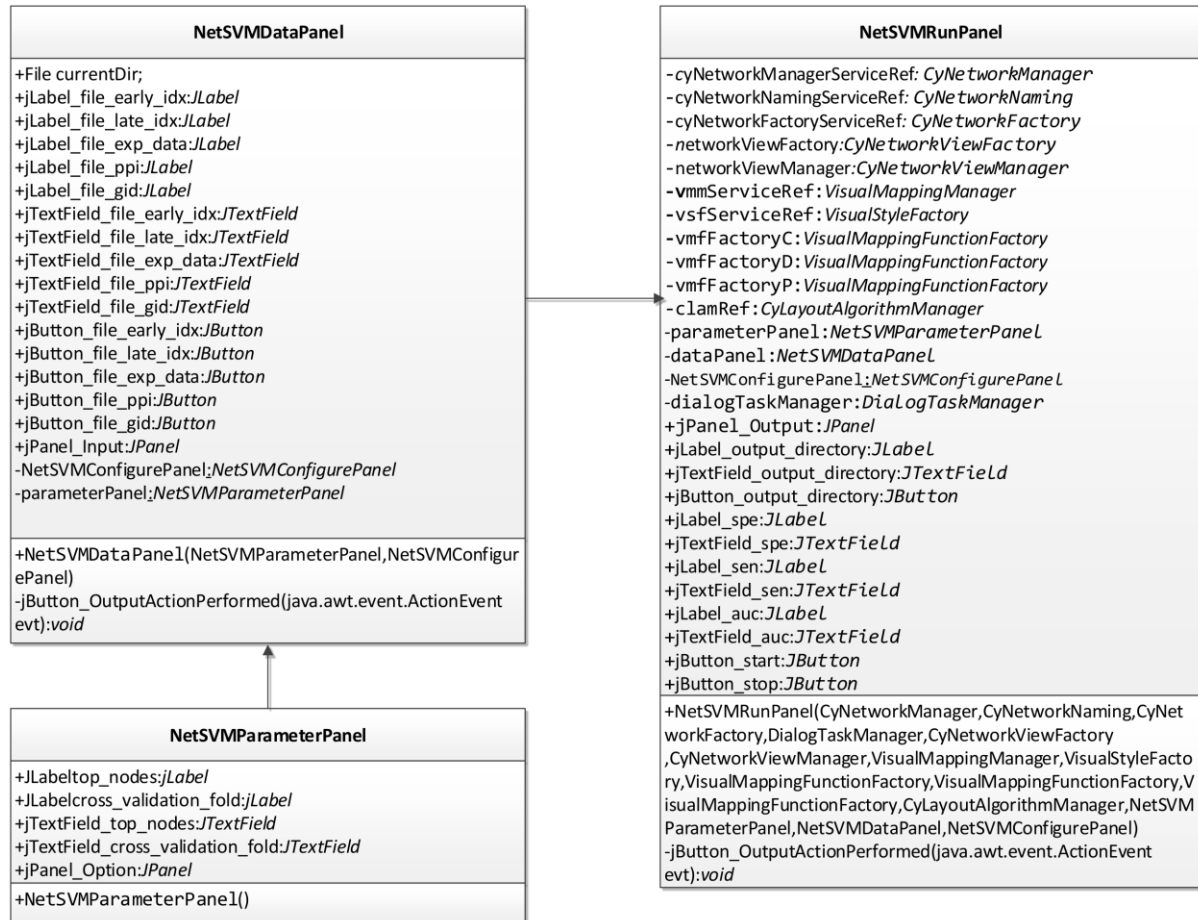

Supplement: S1 Fig — (PDF) [file pone.0170482.s001.pdf]

**S2 Fig. The class diagram of the CyNetSVM bundle application**

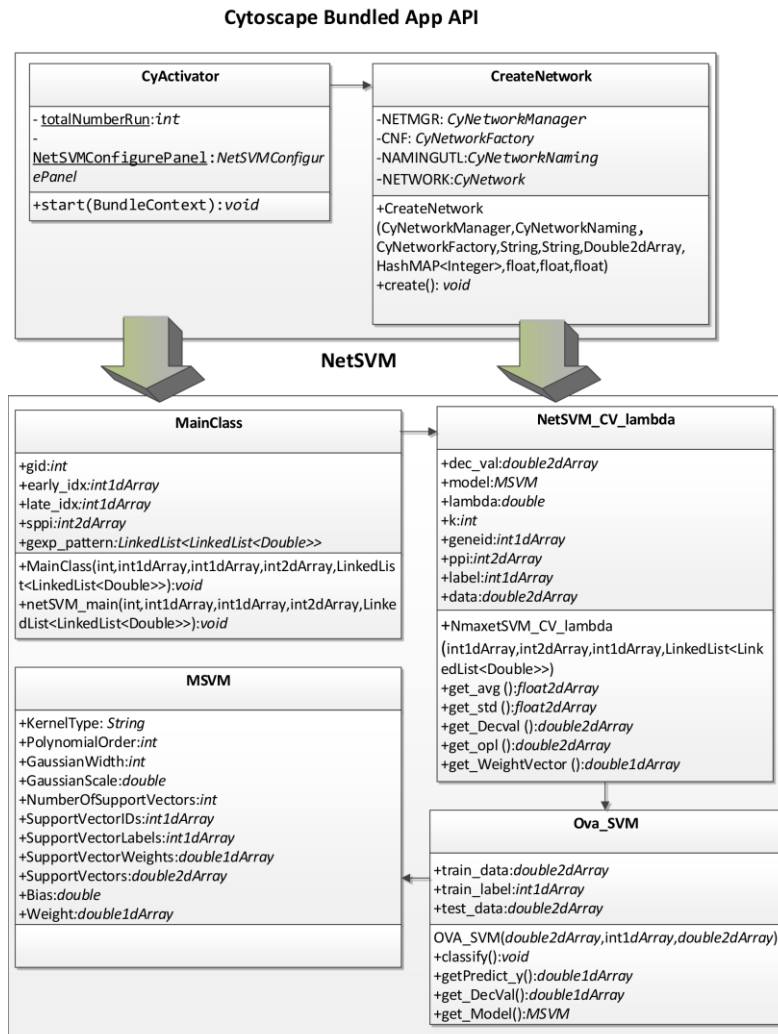

Supplement: S2 Fig — (PDF) [file pone.0170482.s002.pdf]
